# Response to Infant Cry in Clinically Depressed and Non-Depressed Mothers

**DOI:** 10.1371/journal.pone.0169066

**Published:** 2017-01-03

**Authors:** Gianluca Esposito, Nanmathi Manian, Anna Truzzi, Marc H. Bornstein

**Affiliations:** 1 Department of Psychology and Cognitive Science, University of Trento, Rovereto, Trentino, Italy; 2 Division of Psychology, School of Humanities and Social Sciences, Nanyang Technological University, Singapore; 3 Westat, Rockville, Maryland, United States of America; 4 Child and Family Research, *Eunice Kennedy Shriver* National Institute of Child Health and Human Development, Bethesda, United States of America; Harvard Medical School, UNITED STATES

## Abstract

**Background:**

Bowlby and Ainsworth hypothesized that maternal responsiveness is displayed in the context of infant distress. Depressed mothers are less responsive to infant distress vocalizations (cry) than non-depressed mothers. The present study focuses on acoustical components of infant cry that give rise to responsive caregiving in clinically depressed (*n* = 30) compared with non-depressed mothers (*n* = 30) in the natural setting of the home.

**Methods:**

Analyses of infant and mother behaviors followed three paths: (1) tests of group differences in acoustic characteristics of infant cry, (2) tests of group differences of mothers’ behaviors during their infant’s crying, and (3) tree-based modeling to ascertain which variable(s) best predict maternal behaviors during infant cry.

**Results:**

(1) Infants of depressed mothers cried as frequently and for equal durations as infants of non-depressed mothers; however, infants of depressed mothers cried with a higher fundamental frequency (*f*0) and in a more restricted range of *f*0. (2) Depressed mothers fed, rocked, and touched their crying infants less than non-depressed mothers, and depressed mothers were less responsive to their infants overall. (3) Novel tree-based analyses confirmed that depressed mothers engaged in less caregiving during their infants’ cry and indicated that depressed mothers responded only to cries at higher *f*0s and shorter durations. Older non-depressed mothers were the most interactive with infants.

**Conclusions:**

Clinical depression affects maternal responsiveness during infant cry, leading to patterns of action that appear poorly attuned to infant needs.

## Introduction

Mothers’ sensitive responding to their offspring is a signal marker of proper caregiving that has significant implications for many areas of child development [1,2]. Caregiver sensitive responsiveness was first formulated in the context of attachment theory, and germane to the study presented here Bowlby [3] and Ainsworth [4,5] hypothesized that the principal function of maternal sensitive responsiveness is to provide a safe haven for infants in the context of distress [6,7,8]. Maternal sensitive responsiveness encompasses distinct components of perceiving infant signals, interpreting them, and responding to them promptly, appropriately, and contingently via vocal, facial, and tactile channels of communication [9,10,11]. A principal individual-difference factor that interferes with mothers’ abilities to respond sensitively to their infants’ needs is their own depression. Because of her own emotional and cognitive state, the depressed mother is less likely to notice her infant’s signals, to appraise and attribute those signals correctly, and to react to them promptly and appropriately [5,12].

### Depressed Maternal Behavior

Postnatal depression (PND) is a relatively common condition in mothers, affecting typically 10–15% of mothers in high-income countries [13,14] but up to 50% of mothers in low- and middle-income countries [15]. PND has been associated with altered parent-infant interactions [16,17], and specifically with impairments in parental responsiveness to infant cues [18]. Depressed mothers provide their infants with less optimal levels of general stimulation [19,20], and they manifest lower levels of responsiveness in interacting with their infants [21]. Lovejoy, Graczyk, O’Hare, and Neuman [22] meta-analyzed 46 studies linking maternal depression with observed mothering and learned that the strongest relations obtained in children under 1 year of age. PND affects parenting in many different ways, but particularly disrupts processes of attention, attribution, and motivation that are vital parenting capabilities [23,24]. In terms of responsiveness to infant signals, for example, disrupted responsiveness to negative stimuli, such as distress in infant cries, has been found in mothers with PND [25,26] and adults with depression [27]. PND has also been associated with increased risk for childhood cognitive and socio-emotional problems [19,28,29]. Infants of depressed mothers are therefore at-risk developmentally.

### Depressed Maternal Behavior in the Context of Infant Cry

Empirical evidence supports the significance of responsiveness to infant distress signals relative to responsiveness to other signals [6,11]. One of the most salient features of sounds is their fundamental frequency (or *f*0). The *f*0 is the lowest frequency of a sound and corresponds to the pitch of a note. Studies that have experimentally investigated the behavior of depressed mothers during their infant`s cry show that, compared to non-depressed mothers, depressed mothers display less responsiveness to changes in fundamental frequency (*f*0, basic pitch of the cry), *f*0 is less arousing and less salient to them, and they are less likely to respond to infant cry with caregiving [25]. Schuetze and Zeskind [30] also showed that depressed women rate cries as less perceptually salient and less likely to elicit active caregiving. Taken together, these kinds of results suggest that depression alters maternal perceptions of and behavioral reactions to infants.

Physiologically, some variants in genes related to affiliation and social behavior, for example the gene coding for the oxytocin receptors, which is present in the population in several allelic variants, have been found to contribute to individual social sensitivity and maternal behaviors. Individuals with the G allele in several regions of the OXTR gene (compared with the A allele) show more empathy [31]. Riem, Pieper, Out, Bakermans-Kranenburg, and van IJzendoorn [32] studied the role of oxytonergic system gene (OXTR) in physiological reactivity to repeated cry sounds, finding that the GG genotype in the rs53576 region of the OXTR gene is positively related to responsive parenting. They measured heart rate in 40 healthy females without children during the presentation of three episodes of infant cry. Participants with the (presumably) more responsive variant of OXTR GG displayed more pronounced physiological reactivity to cries, except when they showed more symptoms of depression. These results suggest that the OXTR genotype mediates maternal physiological reactivity to infant crying and that depressive symptoms suppress the effect of the OXTR GG genotype. Similarly, employing functional Magnetic Resonance Imaging, Laurent and Ablow [33] investigated depression-related differences in primiparous mothers’ neural response to their own infant’s distress vocalizations. Depressed mothers showed a disturbance of neural networks involved in emotional responses and regulation of responsive parental behaviors. Specifically, non-depressed mothers activate a distributed network of paralimbic and prefrontal regions to their own infant’s cry vis-à-vis control sounds, whereas depressed mothers fail to show such differential activation. Depressed mothers, compared to non-depressed mothers, show significantly smaller striatal (caudate, nucleus accumbens) and medial thalamic activity [34]. Additionally, depressed compared to non-depressed mothers activate uniquely to “own” infant cry more than to “other” infant cry in occipital fusiform areas [33]. In short, depressive symptoms might disrupt parenting in part by undermining attention to child input and reducing child-oriented motivation, goals, and expressions of positive emotion [12].

### Expressions of Distress in Infants of Depressed Mothers

Only a few studies have specifically investigated expressions of distress in infants of depressed mothers. Milgrom, Westley, and McCloud [35] found that diurnal variations in crying patterns of infants of depressed and non-depressed mothers were similar. However, infants of depressed mothers cried significantly more in total per day than infants of non-depressed mothers at 3, but not at 6, months. These results could not be explained by differences in infant temperament. Maternal depression likely contributes to infant crying at 3 months of age [35]. Field and Diego [36] suggested that infants of depressed mothers have tonic differences in parasympathetic responses that may index corresponding socioemotional dysregulation, and when Jones [37] examined newborns’ behavioral and physiological reactions to the cry of another infant (labeled a reactive cry) and to a simulated sound (the control condition), newborns of depressed mothers were found to have lower basal parasympathetic tone (cardiovascular activity, vocal distress, and regulatory behaviors). In addition, infants of depressed mothers responded with less vocal distress to the cry sounds of another infant. Taken together, these findings suggest that infants of depressed mothers show altered, possibly dysregulated, behavioral and physiological patterns during socio-emotional situations in comparison to newborns of non-depressed mothers.

### Aims of the Present Study

The present study contributes to the literatures on the behavior of infants of depressed mothers and parenting in depressed mothers, specifically focusing on infant crying and aims to investigate the interplay between infants’ behavior characteristics and maternal individual characteristics for maternal behavioral responsiveness. We studied the acoustic and behavioral components of infants`cries as well as behaviors of clinically depressed (not just symptomatic) mothers compared with non-depressed mothers in the natural setting of the home during their infant`s crying. To isolate effects to parenting, we controlled for possible confounding factors, such as infant age, gender, and birth order; mother education, age, and working status; and father residence in the household.

## Methods

### Participants and Selection Criteria

Following procedures used in Manian and Bornstein [17], mothers (>20 years) were initially recruited through mass mailings, women’s groups, and newspaper advertisements from the Washington DC metropolitan area. Between 4 and 20 weeks postpartum, mothers completed the Beck Depression Inventory (BDI-II [38]), a 21-item (4-point scale ranging from 0–3) self-report measure developed as an indicator of the presence and degree of depressive symptoms consistent with the DSM-IV (and not as an instrument for specifying a clinical diagnosis). Women with low (1–7) and high scores (>12) were then recruited to participate between 3 and 5 months postpartum in the Structured Clinical Interview for DSM-IV Axis I Disorders (SCID-I [39]). Of mothers with high BDI scores, 30 were diagnosed as having had major or minor depression within the then 5-month lifetime of the child and were included in the depressed group. Of mothers with low-BDI scores, mothers not diagnosed with any depressive disorder were selected into the non-depressed group. Thus, 30 clinically depressed (70% male infants, 53.3% firstborns) and 30 sociodemographically matched non-depressed mothers (67.7% male infants, 60% firstborns) provided data. Table 1 gives sociodemographic statistics for the two groups. All infants were term, healthy, singletons with no known genetic disorders or birth complications, and they averaged 157.40 days (*SD* = 8.39) of age at the observation. Mothers averaged 31.85 years (*SD* = 4.02); their ethnic distribution included 60.0% European American, 16.7% African American, 10.0% Latin American, 10.0% Asian American, and 3.3% mixed or other ethnicity; 20% had partial college or less, 50.0% had completed college, and 30.0% had completed university graduate programs. There were no differences in sociodemographic comparisons between the two groups. The study was approved by the IRB of the *Eunice Kennedy Shriver* National Institute of Child Health and Human Development and was conducted according to the principles expressed in the Declaration of Helsinki. Written informed consent was obtained from the participants.

**Table 1:** Sociodemographic Characteristics of Participants.

	Depressed	Non-Depressed	*t/*χ^2^	*p*	95% CI
**Infant**					
Age (days)	159.28 (9.28)	155.34(6.86)	*t*(58) = 1.80	0.08	[-0.48 8.46]
Gender (% girls)	27.6	26.9	χ^2^(1) = 0.01	0.92	-
Birth order (% firstborn)	53.3	70.4	χ^2^(1) = 2.36	0.12	-
**Mother**					
Age at infant birth (years)	32.22 (4.41)	31.42 (3.06)	*t*(58) = 0.72	0.48	[-1.38 2.91]
Education^a^	6.13 (.76)	6.15 (.44)	*t*(58) = -0.08	0.94	[-0.4 0.37]
Ethnicity (% European American)	63.3	70.4	χ^2^(1) = 0.38	0.54	-
Hollingshead (1975) SES	54.76(6.75)	53.26 (4.47)	*t*(58) = 0.48	0.63	[-4.78 7.80]
Lives with biological father of child (% yes)	100	100	--	--	-

*Note*. *Ms* (*SDs*) are reported, unless otherwise specified.

^a^ Maternal education was scored on a 7-point scale: 1 = less than 7^th^ grade; 2 = 7^th^, 8^th^, 9^th^ grade; 3 = 10^th^ or 11^th^ grade; 4 = high school graduated or GED; 5 = partial college or specialized training; 6 = standard college or university graduate; 7 = graduate professional training.

### Procedures

Infants and mothers were visited in their home, and mothers were asked to behave as they normally would and to ignore the presence of the researcher. Infants and mothers were audio/video recorded continuously for a minimum of 50 min; 50 min of observation falls within the optimal time frame for observations according to Holden and Miller’s [40] meta-analysis of parenting.

Infants were awake 99.22% of the session on average, and no group difference was found, *t*(58) = .49, *p* = .63. Mothers were in view of their infants 92.72% of the observation session on average, and no group difference was found, *t*(58) = 1.07, *p* = .29. No minimum number of cries or minimum time spent in crying were required for the infant and the mother to be included in the study. However, the frequency and the duration of cries did not differ between the two groups (Frequency: *t*(58) = -.60, *p* = .55; Duration: *t*(58) = -.59, *p* = .44). To assess the ecological validity of the observations, infants’ and mothers’ behaviors during the observation were rated on 3-point scales where 1 = *very comfortable/typical* and 3 = *not comfortable/ typical*. On average, mothers reported that they were comfortable (*M* = 1.49, *SD* = .26) during the observation and rated their infants’ (*M* = 1.26, *SD* = .54) and their own (*M* = 1.33, *SD* = .43) behaviors as typical. No group differences were found for the three measures; respectively, and the *t* (58) values were -.70, *p* = .49; .66, *p* = .51; -.32, *p* = .75.

### Acoustic Analysis of Infant Cries

Infant distress vocalizations were quantified and acoustically analyzed from the audio records. Specifically, the frequency and total duration of all infant cries (defined as expressed vocalization of distress > 1 s without any pauses between cry utterances > 10 s) were calculated, and their acoustic parameters were quantified. Infant cries were first digitized, and then digital signal processing and acoustic measurements were accomplished using Praat software [41]. The sampling rate was 44,100 Hz, and the signal was low-pass filtered at 10,000 Hz [42]. The minimum and maximum fundamental frequency (*f*0) values were initially set to 200 and 700 Hz, respectively. These values are consistent with data on typical infant cries [43], and no cry epochs were excluded because all *f*0s fell within the 200–700 Hz band. A long-term average spectrum (LTAS) was applied to provide spectral information for the crying epochs. LTAS has been helpful in discriminating the acoustic characteristics of different categories of infant cries [44,45,46,47]. For all epochs of crying, the First Spectral Peak (FSP) of the LTAS was obtained. FSP is the frequency value (in Hz) of the first amplitude peak across the LTAS. It is an estimate of the average *f*0 of epochs of crying [47]. In addition to *f*0, pitch maximum (*f*0 max = highest level of the FSP) and pitch variability (*f*0 range = range of *f*0 across the cry epoch) were analyzed.

### Behavioral Coding of Maternal Behaviors

Audiovisual records were also coded for mothers’ behaviors. Mothers’ behaviors from the onset to the offset of infants’ crying episodes were coded. An adapted version of the coding scheme developed by Bornstein [48] was applied to code maternal behaviors. The cumulative duration of each behavior divided the total duration the infant cried was determined for the following maternal behaviors: (1) Look was gaze directed to the infant; (2) Verbal was vocalizations, words, and speech-like sounds; (3) Distract was physically moving the infant or an object so that the baby could see or touch it; (4) Hold was initiating or continuing to hold the baby either while sitting or standing/walking; (5) Feed was giving the infant liquid or solid foods by cup, bottle, breast, or spoon; (6) Rock was swaying the infant back and forth at a steady pace; (7) Touch was tactily stimulating the infant (patting or stroking); (8) Kiss/Smile was kissing or smiling at the infant; and (9) No Action was the mother not performing any of the above. Two coders were trained to reliability on each behavior and maintained reliability throughout coding. *Kappa* (*k* [49]) was used to assess reliability of the inter-coder agreement and was calculated on 33% of the sample for all 9 variables (*ks* range = .91 to .95). No differences emerged for the *k*s calculated on the variables that were more prevalent during the observations, such as Look or Verbal, compared to *k*s calculated on those less prevalent, such as Kiss. From the first 8 maternal variables, which represented generally favorable caregiving behaviors, we created an index score for aggregated maternal behaviors that occur during infant cry, the mean *z*-score of the relative durations of possible maternal caregiving behaviors during their infant’s cry.

### Covariates

Covariates included variables that were not controlled for in participant selection or observational procedures and so constitute variation possibly associated with sampling error. The candidate covariates were infant age, gender, and birth order; mother education, age, and working status; and father co-residence in the household.

### Analytic Plan

Analyses of mother and infant behaviors followed three main paths: (1) tests of group differences for infant crying (frequency, duration, and acoustical characteristics: *f0*, *f0* max, and *f0* range), (2) tests of group differences for mother behaviors during infant crying by means of group comparisons and general linear models, and (3) tree-based modeling to ascertain the variable(s) that best predict maternal behavior during infant cry. The clinically depressed versus non-depressed comparison was always of primary interest. However, effects of possible covariates on mother and infant behaviors were also evaluated. Because no main or interaction effects emerged for these covariates, final tests were conducted comparing the two whole groups using *t*-tests to maximize power to detect an effect. A power analysis was computed to determine whether the sample size of 60 was sufficient to detect small-sized effects in a *t*-test with two independent-groups design. With an effect size *d* = .2, α = .05, and *N* = 60, the power was .92, indicating adequate power to detect small, medium, or large effects [50,51]. We report Cohen’s [52] *d* as a measure of effect size for all tests of group comparisons.

## Results

### Preliminary Analysis

Prior to data analysis, distributions of the dependent variables and potential covariates as well as correlations among the infant and maternal behaviors were examined. No transformations were necessary. The correlation between infant cry duration and *f*0 max was not significant (*r* = -.18, *p* = .19). Correlations among the maternal variables ranged between *r* = -.48 and *r* = .43. As expected, some maternal variables were correlated; however, these variables shared little common variance (range = 19% to 23%), and so we treated them independently.

### Group Comparisons for Infant Cry

Table 2 presents the means, standard deviations, and tests of group difference for acoustic parameters of infant distress vocalizations. No statistically significant group differences emerged for the frequency, total duration, and *f*0 max of infant cry. However, significant differences emerged for *f*0 and the *f*0 range. Infants of clinically depressed mothers cried with higher *f*0 and in a more restricted *f*0 range than infants of non-depressed mothers.

**Table 2:** Acoustic Characteristics of Infant Distress Vocalizations: Group Comparisons.

	Depressed	Non-Depressed	*t*(58)	*p*	95% CI
Frequency	10.44 (11.67)	11.06 (8.00)	-0.60	0.55	[-4.58 2.47]
Duration (s)	83.65 (198.48)	105.19 (182.25)	-0.59	0.44	[-7.97 18.26]
*f0*	480.56 (21.78)	442.38 (30.44)	4.43	4.6e-05, *d* = 1.15	[20.95 55.61]
*f0* max	646.90 (64.49)	623.23 (59.78)	1.48	0.14	[-8.28 55.63]
*f0* range	257.63 (75.39)	298.94 (71.64)	-2.06	0.04, *d* = .53	[-81.32–1.29]

*Note*. *Ms* (*SDs*) are reported. *f*0 in Hz.

### Group Comparisons in Maternal Behaviors during Infant Cry

Table 3 presents the means, standard deviations, and tests of group differences for the relative durations of mothers’ behaviors during their infant’s crying. No statistically significant group differences emerged for the duration of mothers’ looks, verbal, distracts, holds, and kisses/smiles. Significant differences emerged for mothers’ feeding, rocking, and touching their infants, and failure to act. Non-depressed mothers fed, rocked, and touched their infants for relatively more time than clinically depressed mothers. Furthermore, a significant difference emerged for the index score for maternal behaviors occurring during infant cry: Non-depressed were overall more active than clinically depressed mothers.

**Table 3:** Relative Durations (s) of Maternal Behaviors during Infant Cry: Group Comparisons.

	Depressed	Non-Depressed	*t*(58)	*p*	95% CI
Look	49.84 (33.78)	50.61 (18.92)	-0.10	0.99	[-16.57 15.03]
Verbal	34.62 (22.94)	44.87 (30.14)	-1.52	0.14	[-23.80 3.30]
Distract	35.25 (29.10)	40.20 (25.00)	-0.68	0.50	[-19.48 9.58]
Hold	32.35 (32.19)	31.45 (34.81)	0.10	0.92	[-17.24 19.05]
Feed	4.06 (11.97)	15.79 (25.94)	-2.32	0.02, *d* = -.61	[-21.86–1.60]
Rock	3.71 (3.80)	9.29 (12.24)	-2.02	0.048, *d* = -.52	[-11.11–0.04]
Touch	14.10 (14.48)	29.12 (24.40)	-2.77	0.008, *d* = -.72	[-25.86–4.16]
Kiss/Smile	1.94 (5.18)	.89 (2.44)	1.19	0.24	[-0.72 2.81]
No Action	21.43 (36.15)	4.48 (13.97)	2.52	0.015, *d* = .65	[3.48 30.42]
Index Score	-.12 (.44)	.13 (.26)	-3.16	0.003, *d* = -.82	[-0.45–0.10]

*Note*. *Ms* (*SDs*) are reported (%s). Index score for maternal behaviors occurring during infant cry is calculated as the mean *z*-score of the relative durations of all possible maternal behaviors during their infant’s cry.

### General Linear Model to Investigate Maternal Behaviors’ Determining Factors

A General Linear Model (GLM) was performed with the maternal caregiving index score as the dependent variable, maternal group condition as a between-subjects factor (depressed vs non-depressed), and *f*0, cry duration, and maternal age as covariates. A significant main effect of the group condition was found. Mothers with a diagnosis of depression showed a lower caregiving index compared to non-depressed mothers, *F*(1,41) = 8.79, *p* = 0.005. No other significant main effect or interaction effect was found for the covariates.

### Tree-Based Models to Identify Maternal Characteristics during Infant Cry

To determine which maternal demographics (clinical diagnosis and age) and infant cry characteristics (frequency, duration, and acoustical characteristics of *f0*, *f0* max, and *f0* range) were operative in prompting maternal caregiving (index score), we coded and then employed recursive partitioning, specifically regression tree models [53] in an exploratory way. Recursive partitioning explores the structure of a data set while developing easy-to-visualize decision rules for predicting a continuous (regression tree) outcome. Regression tree or tree-based models consist of two main steps: growing (exploring relations among variables) and pruning (minimizing overfitting the data). That is, first, the model explores all possible relations among variables and, second, evaluates which values of the independent variables stratify the dependent variable in sample pairs that differ statistically. Tree-based models provide information about the (1) hierarchy of importance of independent variables in explaining the distribution of data points of the dependent variable and (2) which value (“node”) of the independent variable subdivides (“splits”) the dependent variable in sample pairs that differ statistically. Using the rpart package of statistical software R (ver. 3.1–52), the set of parameters used for the splits was: (1) a node must have at least 30 data-point pairs to be considered for a split, (2) at least 20 data-point pairs were required for each terminal node, and (3) surrogate splits were permitted to allow for missingness. The complexity parameter was set equal to zero to allow each tree to grow to its full size. Then, the tree was pruned (resulting in the optimal tree) to remove branches containing nodes with *t*-values greater than 1.64 (α = 05).

**Fig 1:**
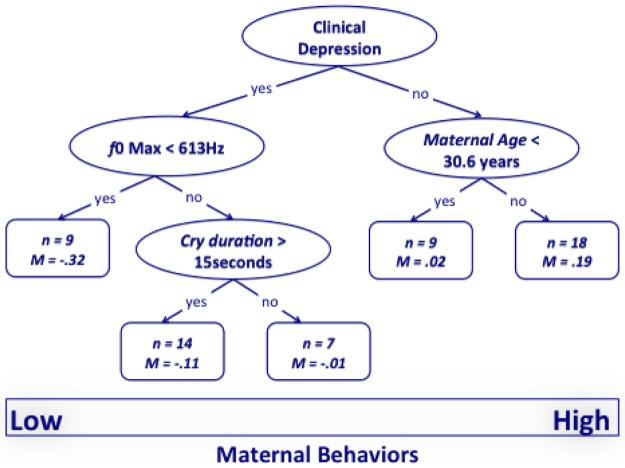
The optimal tree that predicts the index score created to quantify maternal behavior during infant crying. The bottom rectangle shows the distribution of the index score of maternal caregiving from lower (left) to higher (right). The values in oval leaves of the tree refer to the condition of the independent variable that statistically divides the distribution of the dependent variable. Below each oval leaf, the indications ‘‘yes” or ‘‘no” refer to whether or not the condition is met. Each leaf is divided in two sub-leaves. The terminal leaves (quadrangles) represent subgroups that cannot be further subdivided. The *n* value in the terminal leaves represents the size of the group, and *M* is the mean value of the group for the dependent variable.

Fig 1 shows the optimal tree that predicts the index score created to quantify mothers’ responsive behavior during their infant’s crying. The variable that best explained the distribution of the index score of maternal behaviors during their infant’s crying was the clinical diagnosis of the mother. The key role of clinical diagnosis in explaining maternal behavior is consistent with the GLM results. Clinically depressed mothers engaged in less caregiving overall during their infant’s crying. Within the clinically depressed group, moreover, infants who cried at a higher *f*0 (*f*0 max > 613 Hz) had mothers who were more responsive. For infants who cried at a lower fundamental frequency (*f*0 max < 613 Hz), the next level of the tree-model shows that clinically depressed mothers were less responsive to longer duration (> 15 s) cries. At the high caregiving end of the tree, non-depressed mothers’ responsiveness during infant cry related primarily to maternal age (among the non-depressed, older mothers were more responsive than younger mothers). Table 4 shows *Ms* and *SD*s of the maternal index score in the different categories (depressed vs non-depressed mothers, older vs younger mothers, high vs low *f*0 max, long vs short cry duration).

**Table 4:** Combined Group Differences in Maternal Caregiving Index Score.

Group	Maternal Age	*f*0 Max	Cry Duration	Maternal Behavior	Index
Depressed	Old	Low	Long	-0.657	0.150
Depressed	Old	Low	Short	-0.188	0.458
Depressed	Young	High	Short	-0.140	0.207
Non Depressed	Old	High	Short	-0.132	0.081
Depressed	Old	High	Short	-0.100	0.699
Depressed	Old	High	Long	-0.093	0.315
Depressed	Young	Low	Short	-0.092	0.545
Depressed	Young	Low	Long	-0.083	0.092
Non Depressed	Young	High	Long	-0.076	0.150
Depressed	Young	High	Long	-0.074	0.177
Non Depressed	Old	Low	Short	-0.024	0.249
Non Depressed	Young	Low	Long	0.075	0.141
Non Depressed	Young	Low	Short	0.158	0.271
Non Depressed	Old	Low	Long	0.171	0.245
Non Depressed	Old	High	Long	0.195	0.184
Non Depressed	Young	High	Short	0.399	0.346

*Note*. *Ms* and *SDs* are reported. Index score for maternal behaviors occurring during infant cry is reported for each combined group: depressed vs non-depressed; old vs young; low vs high *f*0; long vs short cry duration. Lower index scores represent poorer maternal behaviors. Combined groups are in ascending order for Maternal Behavior Index score, from the lowest to the highest.

## Discussion

Given the acknowledged survival signal function of infant crying [54], universality of responsiveness to infant distress is widely accepted. However, maternal depression interrupts sensitive responsiveness and is an established predictor of parenting problems and poor child outcomes [55,22,17,28,56,57]. This is the first study, to our knowledge, to specifically investigate maternal behavior in the context of infant crying, comparing samples of mothers with clinical depression to non-depressed mothers. Different from studies based on samples with depressive symptomatology, our sample was composed of mothers with clinical depression, diagnosed according to DSM-IV criteria. We found that infants of depressed mothers, during ~1 hour of naturalistic observation, cried as many times and for as long as infants of non-depressed mothers. This result agrees with a previous finding from Milgrom and colleagues [58], showing that diurnal variation in crying patterns of infants of depressed and non-depressed mothers do not differ. However, infants of depressed mothers cried at a higher fundamental frequency and in a more restricted range of variability of *f*0, indicating higher levels of more consistent distress. To our knowledge, this is also the first study to identify and assess the unique acoustic characteristics of cries of infants of clinically depressed mothers. We hypothesize that two sources converge to bring about this result. The first is physiological. Cry *f*0 depends on the flow of air through the vocal cords, and because the lower vocal tract is associated with autonomic nervous system arousal, different *f*0 index differential autonomic nervous system activation. Indeed, infant cries with higher *f*0 are associated with distressing situations and with biological risks of diverse kinds (e.g., Cri du Chat syndrome, asphyxia, brain damage, etc.) and are perceived by parents as more negative and representing a higher distress [59]. Infants of depressed mothers have tonic differences in parasympathetic responses and a lower basal parasympathetic tone (cardiovascular activity, vocal distress, and regulatory behaviors) compared to infants of non-depressed mothers [36,37]. Such differential autonomic activation results in higher cry *f*0.

The second source of infants of depressed mothers expressing a higher level of distress in their crying may be interactional. Cry is one of the few social signals that an infant can perform, and cry characteristics carry important information for a caregiver to know and understand. Different cry *f*0 represent distinct levels of infants’ distress and are related to distinct caregiving behaviors and interactions [60,61]. Normatively, prompt maternal behaviors to infant cry effectively address expressed distress in infants. However, if the communication system between mothers and infants is disrupted (because of a depressed caregiver’s atypical reactions) infants may adapt their communicative signals to elicit an adaptive parental response to their needs by increasing their expressed distress.

Here, we found that depressed and non-depressed mothers show many of the same behaviors, but clinically depressed mothers show significantly less feeding, rocking, and touching and less caregiving to their infants’ cries overall. In other words, clinically depressed mothers are less behaviorally responsive to their crying infants (and in specific ways) than are non-depressed mothers. Dix and Meunier [12] reviewed a large number of studies investigating several features of maternal depression and suggested several possible processes underlying behavioral differences found in depressed vs non-depressed mothers. They interpreted depression symptoms starting from an action-control theory that postulates that human actions emanate from an internal system which promotes the achievement of moment-to-moment goals. Following their proposed model, the lower levels of feeding, rocking, and touching and failure to act in clinically depressed mothers may be explained by the failure of that internal system to generate behavioral responses of either a cognitive nature, such as goal setting, self-focused attention, and negative evaluations of their infants or of their own self, or an emotional nature, such as negative emotions and attitudes. To respond adaptively, we need to act and to act appropriately. Clinically depressed mothers may be non-interactive overall for relatively more time than non-depressed mothers because depressive symptoms (i.e., self-oriented goals and attention, negative attitudes) impede choosing the right behavior to perform resulting in the selection of ineffective responses and fewer response options in general [12]. In addition, mothers with postpartum depression are more likely to show reduced sensitivity, decreased neural activation, as well as more intrusive or withdrawn behaviors in interaction with their infants [62].

Furthermore, we found that younger non-depressed mothers tend to be less responsive than older mothers. A considerable psychological literature supports age-related differences in maternal positive caregiving [63,64]. Our findings accord with that literature.

Our analyses and tree-based deconstruction, which corroborated the GLM findings and added more information allowing us to gain a deeper view of the possible underlying mechanisms, revealed that clinically depressed mothers engage in fewer overall caregiving behaviors when their infants cry. Furthermore, depressed mothers tend to respond only to cries at higher *f*0. Given the special salience of high-frequency sounds and their effectiveness in capturing attention, it could be that depressed mothers need strong and specific stimuli to elicit responsive behaviors. It could be that infants’ increasing cry *f*0 is an adaptation to the diminished baseline responsiveness of their depressed mothers. This interpretation would imply that a higher *f*0 cry, either by individual predisposition or as a learned adaptation to the environment, marks resilience in infants exposed to a risk environment. Further studies are needed to distinguish these possible interpretations. That said, growing evidence indicates that the effects of depression on parenting vary depending on many parent- and family-level factors [65,66].

Infant cry characteristics in the postpartum period vary by maternal depression status. However, differences in infant cry characteristics may be also moderated by genetic predisposition or environmental factors before birth. Future studies should assess shared genetic characteristics [67,68] and experiences during fetal environment to investigate how they interact with maternal behaviors in shaping infant cry. Other maternal characteristics, such as sensitiveness or emotional availability, could also be taken into account in subsequent studies to obtain a broader view of the role maternal behaviors play in mother-infant interactions. The maternal variable Hold merits some additional consideration. Hold was defined as initiating or continuing to hold the baby either while sitting or standing/walking. However, Esposito and colleagues [69]showed that maternal holding while sitting and maternal holding while walking and carrying have two specific and distinct effects on babies. Holding the baby while walking has a calming effect which is not present when holding the baby while sitting. Holding while sitting and holding while carrying may be differently performed by depressed and non-depressed mothers, although both groups may perform the two behaviors for the same amounts of time; therefore, future studies might investigate this difference by focusing on simply holding versus holding-and-carrying. Our results must also be seen as limited in their generalizability, given that our non-depressed and depressed groups consisted of relatively educated mothers at relatively low risk for other psychosocial adversities.

## Conclusion

Using a dyadic approach to study infant and mother behaviors in the context of crying, we found that clinically depressed and non-depressed mothers of 5-month-olds differed in their caregiving. Notably, even in relatively advantaged groups, clinically depressed mothers showed less interactive involvement to their infants’ distress vocalizations. Whereas non-depressed mothers showed higher levels of caregiving during their infants’ crying, depressed mothers tended to respond only when crying reached higher *f*0. This study advances our understanding of interactional patterns during infants’ expressions of needs, showing that in the context of distress infants of clinically depressed mothers behave differently to infants of non-depressed mothers. On this account, infants of depressed mothers may show differences in behaviors that index socioemotional dysregulation. Parent training is of greater benefit to families of non-depressed than depressed parents [70], perhaps because of depressed mothers’ low attention, affect, and action. Our results further suggest that focused counseling in depressed mothers toward responsiveness might enhance maternal caregiving. However, future studies should also take into account mothers’ degree of depression and how different clinical levels of depression shape maternal responses to their infants.

## References

[1] Bornstein M. H. (2015). Children’s parents In Bornstein M. H. & Leventhal T. (Eds.), *Ecological settings and processes in developmental systems*. *Volume 4 of the Handbook of child psychology and developmental science* (7e, pp. 55–132). Editor-in-chief: Lerner R. M.. Hoboken, NJ: Wiley.

[2] Sroufe L. A., Egeland B., Carlson E., & Collins W. A (2009). *The development of the person*: *The Minnesota study of risk and adaptation from birth to adulthood*. New York: Guilford.

[3] Bowlby J. (1969). Attachment and loss: Vol. 1. Attachment. New York: Basic Books.

[4] Ainsworth M. D. S. (1969). Object relations, dependency, and attachment: A theoretical review of the infant-mother relationship. *Child Development*, 40(4), 969–1025. 5360395

[5] Ainsworth M. D. S., Blehar M. C., Waters E., & Wall S. (1974). *Patterns of attachment*: *A psychological study of the strange situation*. New York: Psychology Press.

[6] Higley E., & Dozier M. (2009). Nighttime maternal responsiveness and infant attachment at one year. *Attachment & Human Development*, 11, 347–363. 19603300 10.1080/14616730903016979 PMC3422632

[7] Leerkes E. M., Blankson A. N., & O’Brien M. (2009). Differential effects of sensitivity to infant distress and non-distress on social-emotional funcitoning. *Child Development*, 80, 762–775. 10.1111/j.1467-8624.2009.01296.x 19489902 PMC2854550

[8] McElwain N. L., & Booth-LaForce C. (2006). Maternal sensitivity to infant distress and nondistress as predictors of infant-mother attachment security. *Journal of Family Psychology*, 20, 247–255. 10.1037/0893-3200.20.2.247 16756400

[9] Ainsworth M. D. S., Bell S. M., & Stayton D. J. (1974). Infant-mother attachment and social development In Richards M. P. (Ed.), *The introduction of the child to the social world* (pp. 99–135). London: Cambridge University Press.

[10] Bornstein M. H., & Tamis-LeMonda C. S. (1989). Maternal responsiveness and cognitive development in children. *New Directions for Child and Adolescent Development*, 1989(43), 49–61. 10.1002/cd.23219894306 2710392

[11] Mesman J., van IJzendoorn M. H., & Bakermans-Kranenburg M. J. (2009). The many faces of the still-face paradigm: a review and meta-analysis. *Developmental Review*, 29(2), 120–162

[12] Dix T., & Meunier L.N. (2009). Depressive symptoms and parenting competence: an analysis of 13 regulatory processes. *Developmental Review*, 29, 45–68.

[13] O'Hara M.W. and Swain A.M. (1996) Rates and risk of postpartum depression—a meta-analysis. *International Review of Psychiatry* 8, 37–54

[14] Paulson J.F. and Bazemore S.D. (2010) Prenatal and postpartum depression in fathers and its association with maternal depression: a meta-analysis. *Jama* 303, 1961–1969 10.1001/jama.2010.605 20483973

[15] Parsons C. E., Young K. S., Rochat T. J., Kringelbach M. L., & Stein A. (2012) Postnatal depression and its effects on child development: a review of evidence from low- and middle-income countries. *Br Med Bull* 101, 57–79 10.1093/bmb/ldr047 22130907

[16] Bigelow A. E., MacLean K., Proctor J., Myatt T., Gillis R., & Power M. (2010) Maternal sensitivity throughout infancy: Continuity and relation to attachment security. *Infant Behavior and Development* 33, 50–60 10.1016/j.infbeh.2009.10.009 20004022

[17] Manian N., & Bornstein M. H. (2009).Dynamics of emotion regulation in infants of clinically depressed and nondepressed mothers. *Journal of Child Psychology and Psychiatry*, 50(11), 1410–1418. 10.1111/j.1469-7610.2009.02166.x 19788549 PMC2844731

[18] Lester B. M., Boukydis C. Z., Garcia-Coll C. T., Peucker M., McGrath M. M., Vohr B. R., et al. (1995) Developmental outcome as a function of the goodness of fit between the infant's cry characteristics and the mother's perception of her infant's cry. *Pediatrics* 95, 516–521 7700751

[19] Bornstein M. H., Arterberry M. E., Mash C., & Manian N. (2011). Discrimination of facial expression by 5-month-old infants of nondepressed and clinically depressed mothers.*Infant Behavior and Development*, 34(1), 100–106. 10.1016/j.infbeh.2010.10.002 21112092 PMC3174100

[20] Murray L., Hipwell A., Hooper R., Stein A., & Cooper P. (1996). The cognitive development of 5-year-old children of postnatally depressed mothers. *Journal of Child Psychology and Psychiatry*, 37(8), 927–935. 9119940 10.1111/j.1469-7610.1996.tb01490.x

[21] National Institute of Child Health and Human Development (NICHD) Early Child Care Research Network, (1999). Child care and mother-child interaction in the first 3 years of life. *Developmental Psychology*, 35, 1399–1413. 10563730

[22] Lovejoy M.C., Graczyk P.A., O’Hare E., & Neuman G. (2000). Maternal depression and parenting behavior: a meta-analytic review. *Clinical Psychology Review*, 20(5), 561–592. 10860167 10.1016/s0272-7358(98)00100-7

[23] Dix T., Moed A., & Anderson E.R. (2014). Mothers’ depressive symptoms predict both increased and reduced negative reactivity: aversion sensitivity and the regulation of emotion. Psychological Science, 25(7), 1353–1361. 10.1177/0956797614531025 24796661

[24] Psychogiou L., & Parry E. (2014). Why do depressed individuals have difficulties in their parenting role? *Psychological Medicine*, 44 *(*7*)*, 1345–1347. 10.1017/S0033291713001931 24128783

[25] Donovan WL, Leavitt LA, Walsh RO. (1998). Conflict and depression predict maternal sensitivity to infant cries. *Infant Behavior and Development*, 21:505–517.

[26] Stein A., Arteche A., Lehtonen A., Craske M., Harvey A., Counsell N., et al. (2010) Interpretation of infant facial expression in the context of maternal postnatal depression. *Infant Behav Dev* 33, 273–278 10.1016/j.infbeh.2010.03.002 20381873 PMC2896481

[27] Young K. S., Parsons C. E., Stein A., & Kringelbach M. L. (2012) Interpreting Infant Vocal Distress: The Ameliorative Effect of Musical Training in Depression. *Emotion* 12, 1200–1205. 10.1037/a0028705 22775126 PMC3518372

[28] Pearson R. M., Bornstein M. H., Cordero M., Scerif G., Mahedy L., Evans J., Abioye A., & Stein A. (2016). Maternal perinatal mental health and offspring academic achievement at age 16: The mediating role of childhood executive function. *Journal of Child Psychology and Psychiatry*, 57, 491–501. 10.1111/jcpp.12483 26616637 PMC4789117

[29] Van Ijzendoorn M. H., Schuengel C., & Bakermans—Kranenburg M. J. (1999) Disorganized attachment in early childhood: meta-analysis of precursors, concomitants, and sequelae. *Developmental Psychopathology* 11, 225–249 10.1017/s0954579499002035 16506532

[30] Schuetze P, Zeskind PS. 2001 Relations between women’s depressive symptoms and percep- tions of infant distress signals varying in pitch. *Infancy*, 2:483–499. 10.1207/S15327078IN0204_06 33451195

[31] Wu N., Zhi L., Su Y. (2012). The association between oxytocin receptor gene polymorphism (OXTR) and trait empathy. *Journal of Affective Disorders*, 138, 468–472. 10.1016/j.jad.2012.01.009 22357335

[32] Riem M. M., Pieper S., Out D., Bakermans-Kranenburg M. J., & van IJzendoorn M. H. (2011). Oxytocin receptor gene and depressive symptoms associated with physiological reactivity to infant crying. *Social cognitive and affective neuroscience*, 6(3), 294–300. 10.1093/scan/nsq035 20400491 PMC3110427

[33] Laurent H. K., & Ablow J. C. (2012). A cry in the dark: depressed mothers show reduced neural activation to their own infant’s cry. *Social Cognitive and Affective Neuroscience*, 7(2), 125–134. 10.1093/scan/nsq091 21208990 PMC3277361

[34] Chase H. W., Moses-Kolko E. L., Zevallos C., Wisner K. L., & Phillips M. L. (2014). Disrupted posterior cingulate—amygdala connectivity in postpartum depressed women as measured with resting BOLD fMRI. *Social cognitive and affective neuroscience*, 9(8), 1069–1075. 10.1093/scan/nst083 23709351 PMC4127008

[35] Milgrom J., Westley D. T., & McCloud P. I. (1995). Do infants of depressed mothers cry more than other infants?.*Journal of Paediatrics and Child Health*, 31(3), 218–221. 7669383 10.1111/j.1440-1754.1995.tb00789.x

[36] Field T., Diego M.A., 2008 Vagal activity, early growth and emotional development. *Infant Behavior and Development*, 31, 36–373. 10.1016/j.infbeh.2007.12.008 PMC2556849 18295898

[37] Jones N. A. (2012). Delayed reactive cries demonstrate emotional and physiological dysregulation in newborns of depressed mothers. *Biological Psychology*, 89(2), 374–381. 10.1016/j.biopsycho.2011.11.011 22155474

[38] Beck A.T., Steer R.A., & Brown G.K. (1996).*Manual for the Beck Depression Inventory-II*. San Antonio, TX: Psychological Corp.

[39] First M.B., Spitzer R.L, Gibbon M., & Williams J.B.W. (2001).*Structured clinical interview for DSM-IV-TR axis I disorders*, *research version*, *non-patient edition (SCID-I/NP)*. New York: Biometrics Research, New York State Psychiatric Institute.

[40] Holden G. W., & Miller P. C. (1999). Enduring and different: A meta-analysis of the similarity in parents’ child rearing. *Psychological Bulletin*, 125, 223–254. 10087937 10.1037/0033-2909.125.2.223

[41] Boersma, P., & Weenink, D. (2005). Praat: Doing phonetics by computer (Version 5.0.06) PC Software Available from http://www.praat.org.

[42] Rautava L., Lempinen A., Ojala S., Parkkola R., Rikalainen H., Lapinleimu H.,& PIPARI Study Group. (2007). Acoustic quality of cry in very-low-birth-weight infants at the age of 1 1/2 years. *Early Human Development*, 83(1), 5–12. 10.1016/j.earlhumdev.2006.03.004 16650947

[43] Rothganger H. (2003). Analysis of the sounds of the child in the first year of age and a comparison to the language. *Early Human Development*, 75, 55–69. 14652159 10.1016/j.earlhumdev.2003.09.003

[44] Esposito G., & Venuti P. (2010). Understanding early communication signals in Autism Spectrum Disorder: A study on perception of cry in toddlers. *Journal of Intellectual Disability Research*, 54(3), 216–223. 10.1111/j.1365-2788.2010.01252.x 20136681

[45] Esposito G., Nakazawa J., Venuti P., Bornstein MH. (2012) Perceptions of distress in young children with autism compared to typically developing children: a cultural comparison between Japan and Italy. *Research in Developmental Disabilities*, 33(4):1059–1067. 10.1016/j.ridd.2012.01.014 22502830 PMC3328100

[46] Esposito G., Rostagno M., del C., Venuti P., Haltigan J. D., & Messinger D. M. (2014). Atypical expression of distress during the separation phase of the strange situation in infant siblings at high risk for ASD. *Journal of Autism and Developmental Disorders*, 44(4), 975–980. 10.1007/s10803-013-1940-6 24026913 PMC3949194

[47] Lin H. C., & Green J. A. (2007). Effects of posture on newborn crying. *Infancy*, 11(2), 175–189.

[48] Bornstein M.H. (2001). *Manual for observation and analysis of infant development and mother-infant interaction* Child and Family Research, Eunice Kennedy Shriver National Institute of Child Health and Human Development, Bethesda, MD 20892.

[49] Cohen J. (1968). Weighted kappa: Nominal scale agreement provision for scaled disagreement or partial credit. *Psychological Bulletin*, 70 (4), 213–220. 19673146 10.1037/h0026256

[50] Faul F., Erdfelder E., Buchner A., & Lang A.G. (2009). Statistical power analyses using G*Power 3.1: Tests for correlation and regression analyses. *Behavior Research Methods*, 41, 1149–1160. 10.3758/BRM.41.4.1149 19897823

[51] Faul F., Erdfelder E., Lang A.-G., & Buchner A. (2007). G*Power 3: A flexible statistical power analysis program for the social, behavioral, and biomedical sciences. *Behavior Research Methods*, 39, 175–191. 17695343 10.3758/bf03193146

[52] Cohen J. (1992). Statistical power analysis. *Current directions in psychological science*, 1(3), 98–101.

[53] Costello T. J., Swartz M. D., Sabripour M., Gu X., Sharma R., & Etzel C. J. (2003). Use of tree-based models to identify subgroups and increase power to detect linkage to cardiovascular disease traits. *BMC Genetics*, 4(1), S66. 14975134 10.1186/1471-2156-4-S1-S66 PMC1866504

[54] Ziefman D. M. (2003) Predicting adult responses to infant distress: adult characteristics associated with percep- tions, emotional reactions, and timing of intervention. *Infant Mental Health Journal* 24,597–612.

[55] Jackson A. P., & Huang C. C. (2000). Parenting stress and behavior among single mothers of preschoolers: The mediating role of self-efficacy. *Journal of Social Service Research*, 26, 29–42.

[56] Reyno S., & McGrath P. (2006). Predictors of parent training efficacy for child externalizing behavior problems: A meta-analytic review. *Journal of Child Psychology and Psychiatry*, 47, 99–111. 10.1111/j.1469-7610.2005.01544.x 16405646

[57] Wang Y., & Dix T. (2013). Patterns of depressive parenting: Why they occur and their role in early developmental risk. *Journal of Family Psychology*, 27(6), 884–895. 10.1037/a0034829 24294931

[58] Milgrom J., Gemmill A.W., Bilszta J.L., Hayes B., Barnett B., Brooks J., Ericksen J., Ellwood D., Buist Anne (2008). Antenatal risk factors for postnatal depression: A large prospective study. *Journal of Affective Disorders*, 108, 147–157. 10.1016/j.jad.2007.10.014 18067974

[59] Lester B. M., and LaGasse L. L. (2008). "Crying" *Encyclopedia of infant and early childhood development*. Academic Press, San Diego, CA, p.p. 332–343.

[60] Out D., Pieper S., Bakermans-Kranenburg M.J., Zeskind P.S., van Ijzendoorn M.H. (2010). Intended sensitive and harsh caregiving resposnes to infant crying: the role of cry pitch and percieved urgency in an adult twin sample. *Chile Abuse & Neglect*, 34, 863–873. 10.1016/j.chiabu.2010.05.003 20889206

[61] Ziefman D.M. (2001). An ethological analysis of human infant crying: answering Tinbergen’s four questions. *Developmental Psychobiology*, 39(4), 265–285. 11745323 10.1002/dev.1005

[62] Pechtel P., Murray L., Brumariu L., & Lyons-Ruth K. (2013). Reactivity, regulation, and reward responses to infant cues among mothers with and without psychopathology: an fMRI review. *Translational Developmental Psychiatry*, 1, 19673. 10.3402/tdp.v1i0.19673 PMC6858056 31737224

[63] Bornstein M. H., & Putnick D. L. (2007). Chronological age, cognitions, and practices in European American mothers: A multivariate study of parenting. *Developmental Psychology*, 43, 850–864. 10.1037/0012-1649.43.4.850 17605519 PMC5827928

[64] Bornstein M. H., Putnick D. L., Suwalsky J. T. D., & Gini M. (2006). Maternal chronological age, prenatal and perinatal history, social support, and parenting of infants. *Child Development*, 77, 875–892. 10.1111/j.1467-8624.2006.00908.x 16942495 PMC5827934

[65] Azak S., & Raeder S. (2013). Trajectories of parenting behavior and maternal depression. *Infant Behavior and Development*, 36, 391–402. 10.1016/j.infbeh.2013.03.004 23603820

[66] Mills-Koonce W.R., Gariepy J-L., Sutton K., & Cox M.J. (2008). Changes in maternal sensitivity across the first three years: are mothers from different attachment dyads differentially influenced by depressive symptomatology? *Attachment and Human Development*, 10(3), 299–317. 10.1080/14616730802113612 18821340

[67] Hyde C. L., Nagle M. W., Tian C., Chen X., Paciga S. A., Wendland J. R., et al. (2016). Identification of 15 genetic loci associated with risk of major depression in individuals of European descent. *Nature Genetics*. Advance online publication. 10.1038/ng.3623 PMC5706769 27479909

[68] Weissman M. M., Wickramaratne P., Nomura Y., Warner V., Verdeli H., Pilowsky D. J., et al. (2005). Families at high and low risk for depression: A 3-generation study. *Archives of General Psychiatry*, 43, 29–36. 10.1001/archpsyc.62.1.29 15630070

[69] Esposito G., Yoshida S., Ohnishi R., Tsuneoka Y., Rostagno M del C., Yokota M., et al. (2013). Infant calming responses during maternal carrying in humans and mice. Current Biology, 23(9), 739–745. 10.1016/j.cub.2013.03.041 23602481

[70] Van Loon L.M.A. van, Granic I. & Engels R.C.M.E. (2011). The role of maternal depression on treatment outcome for children with externalizing behavior problems. *Journal of Psychopathology and Behavioral Assessment*, 33(2), 176–186. 10.1007/s10862-011-9228-7 PMC3105234 21765595

